# Comparative analysis of tissue reconstruction algorithms for 3D histology

**DOI:** 10.1093/bioinformatics/bty210

**Published:** 2018-04-19

**Authors:** Kimmo Kartasalo, Leena Latonen, Jorma Vihinen, Tapio Visakorpi, Matti Nykter, Pekka Ruusuvuori

**Affiliations:** 1 Faculty of Medicine and Life Sciences, University of Tampere, Tampere, Finland; 2 Faculty of Biomedical Sciences and Engineering, Tampere University of Technology, Tampere, Finland; 3 BioMediTech Institute, Tampere, Finland; 4 Fimlab Laboratories, Tampere University Hospital, Tampere, Finland; 5 Faculty of Engineering Sciences, Tampere University of Technology, Tampere, Finland; 6 Faculty of Computing and Electrical Engineering, Tampere University of Technology, Tampere 33101, Finland

## Abstract

**Motivation:**

Digital pathology enables new approaches that expand beyond storage, visualization or analysis of histological samples in digital format. One novel opportunity is 3D histology, where a three-dimensional reconstruction of the sample is formed computationally based on serial tissue sections. This allows examining tissue architecture in 3D, for example, for diagnostic purposes. Importantly, 3D histology enables joint mapping of cellular morphology with spatially resolved omics data in the true 3D context of the tissue at microscopic resolution. Several algorithms have been proposed for the reconstruction task, but a quantitative comparison of their accuracy is lacking.

**Results:**

We developed a benchmarking framework to evaluate the accuracy of several free and commercial 3D reconstruction methods using two whole slide image datasets. The results provide a solid basis for further development and application of 3D histology algorithms and indicate that methods capable of compensating for local tissue deformation are superior to simpler approaches.

**Availability and implementation:**

Code: https://github.com/BioimageInformaticsTampere/RegBenchmark. Whole slide image datasets: http://urn.fi/urn: nbn: fi: csc-kata20170705131652639702.

**Supplementary information:**

Supplementary data are available at *Bioinformatics* online.

## 1 Introduction

Digitalization of pathology has been accelerated by improvements in technology allowing acquisition of whole slide images (WSI) (Ghaznavi *et al.*, 2013; Griffin and Treanor, 2017). Besides computer-aided facilitation of pathologists’ tasks, digital pathology can enable new approaches like 3D histology, where three-dimensional reconstructions of samples are formed *in silico* based on serial sections (Magee *et al.*, 2015; Roberts *et al.*, 2012). While other techniques allow imaging directly in 3D, they are currently incapable of matching the subcellular resolution and throughput of whole slide imaging. Examples of potential applications include construction of data-driven computer models and improved diagnostics of diseases associated with changes in the 3D microarchitecture of tissue. Moreover, 3D histology is compatible with established histopathological interpretation techniques and biochemical assays such as immunohistochemistry or *in situ* hybridization. This raises interesting prospects in view of recent advances in spatially resolved omics (Mignardi *et al.*, 2017; Ståhl *et al.*, 2016). Pairing imaging with genomic, epigenomic, transcriptomic and proteomic data in the spatial context of tissue holds great promise for pathology and other fields (Koos *et al.*, 2015). Taking a step further, this could be performed in 3D to truly probe the relationships between structural and functional features as well as the heterogeneity and interplay between different cell types in tumors, and significant projects are now pursuing these goals (Ledford, 2017; Rusk, 2016). These kind of approaches have already led to the creation of brain atlases (Amunts *et al.*, 2013; Johnson *et al.*, 2010; Lein *et al.*, 2007). Such high-dimensional data also represent an exciting challenge for new ways of scientific visualization based e.g. on virtual reality techniques (Calì *et al.*, 2016; Ledford, 2017; Theart *et al.*, 2017).

Despite earlier computational and image acquisition bottlenecks (Roberts *et al.*, 2012), several algorithmic 3D histology solutions were already proposed before the recent developments in digital pathology (Ju *et al.*, 2006; Wang *et al.*, 2015). The key methodological problem is how to accurately register a sequence of 2D images to produce a 3D volume. Simply stacking the images does not result in a coherent volume due to differences between the relative locations and rotation angles of the sections and tissue deformations introduced during embedding and sectioning (Gibson *et al.*, 2013). Algorithms for image registration (Sotiras *et al.*, 2013) constitute the methodological basis of 3D histology. These algorithms are used to sequentially register each image with its neighbors to bring the entire series into alignment (Magee *et al.*, 2015; Wang *et al.*, 2015). Registration is accomplished by estimating transformations relating the images. Rigid transformations only allow translation and rotation of the entire image, while affine transformations are additionally able to model anisotropic scaling. Locally varying transformations, also called elastic models, can compensate for deformations on a local scale. Considering several nearby sections together (Saalfeld *et al.*, 2012) or applying regularization may be needed to obtain smooth, continuous 3D volumes (Casero *et al.*, 2017; Cifor *et al.*, 2011; Gaffling *et al.*, 2015; Ju *et al.*, 2006). After estimating the transformations, they need to be applied to the images via interpolation, which is possibly followed by postprocessing such as 3D visualization. Our focus is on the reconstruction step, which is usually the most difficult and crucial part of the image processing chain. Numerous approaches have been reported, relying on manual alignment (Onozato *et al.*, 2012; Paish *et al.*, 2009), semi-automatic methods using artificial landmarks (Hughes *et al.*, 2013; Rojas *et al.*, 2015) and automated algorithms (Arganda‐Carreras *et al.*, 2010; Braumann *et al.*, 2005; Casero *et al.*, 2017; Cifor *et al.*, 2011; Ju *et al.*, 2006; Magee *et al.*, 2015; Saalfeld *et al.*, 2012; Song *et al.*, 2013; Stille *et al.*, 2013; Xu *et al.*, 2015).

**Fig. 1.**
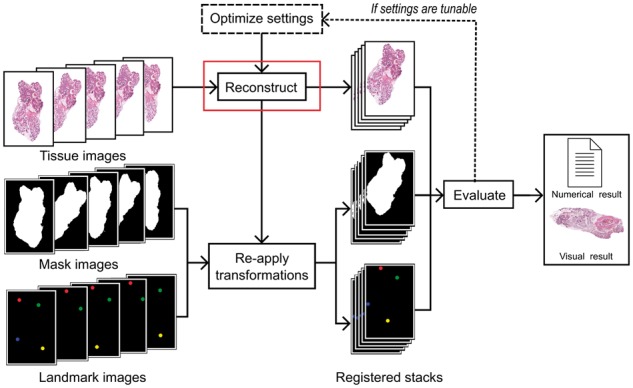
Evaluation framework. A series of tissue images is input to a reconstruction method for registration. The transformations estimated by the method are re-applied to masks defining the tissue region and images containing landmarks. The registered tissue, mask and landmark images are used to evaluate reconstruction accuracy based on numerical metrics and visual examination. Moreover, tunable settings can be optimized. (Color version of this figure is available at *Bioinformatics* online.)

Despite the widely acknowledged need for objective assessment of algorithms (Meijering *et al.*, 2016), an evaluation of modern computational methodology for 3D histology is lacking. Moreover, the common practice of relying only on visual inspections or a single indirect metric is insufficient (Rohlfing, 2012). The previous comparison of algorithms was published a decade ago and only included three basic approaches (Beare *et al.*, 2008). We have previously demonstrated a framework (Kartasalo *et al.*, 2016) based on a panel of indirect metrics and manually annotated landmarks allowing direct quantification of reconstruction accuracy (Rohlfing, 2012). In this study, we applied an extended version of the framework (see Fig. 1) to address the problem of comparing algorithms for 3D histology. As the basis of our evaluation, we used two WSI datasets representing two different tissue types. One obstacle complicating both the application and fair comparison of most algorithms is sensitivity to various settings or hyperparameters, which typically have to be selected by the user based on rules of thumb and tuned via trial and error. Encouraged by their recent application in the context of digital pathology, we employed automated hyperparameter selection methods to adjust tunable parameters (Shahriari *et al.*, 2016; Teodoro *et al.*, 2017).

As a baseline, we evaluated three basic methods: a least-squares fit to landmarks (LS), an optimization-based approach (OPT) and a method based on the Scale Invariant Feature Transform (SIFT) (Lowe, 2004). More advanced methods included the Fiji/ImageJ (Schindelin *et al.*, 2012; Schneider *et al.*, 2012) plugins HyperStackReg (HSR), which is an extension of StackReg (Thevenaz *et al.*, 1998), RegisterVirtualStackSlices (RVSS), which is based on bUnwarpJ (Arganda-Carreras *et al.*, 2006), and ElasticStackAlignment (ESA) (Saalfeld *et al.*, 2012), which is part of the TrakEM2 package (Cardona *et al.*, 2012). In addition, we evaluated two commercial tools: Medical Image Manager (MIM) (HeteroGenius Ltd, Leeds, UK) and Voloom (microDimensions GmbH, Munich, Germany). While LS, OPT, SIFT and HSR are based on global transformations, RVSS, ESA, MIM and Voloom use elastic models which make it possible to account for local tissue deformations. For a summary of the evaluated tools, see Supplementary Table S1.

## 2 Materials and methods

### 2.1 Data collection and preprocessing

A murine prostate and a liver were fixed in PAXgene™ (PreAnalytiX GmbH, Hombrechtikon, Switzerland) and formalin, respectively, embedded in paraffin, and cut into serial 5 µm sections. The liver was processed with a laser prior to embedding in order to introduce artificial landmarks into the otherwise homogeneous tissue. Four holes were successfully introduced into the sample. The sections were hematoxylin-eosin (HE) stained and scanned at 20× (pixel size 0.46 µm) to obtain 260 (prostate) and 47 (liver) RGB images. The images were processed in MATLAB R2016b (The MathWorks Inc., Natick, MA, USA) to segment tissue from background and store the results as binary masks.

A total of 2448 landmarks were manually annotated. In the prostatic tissue, four corresponding points preferably at the centers of bisected nuclei were selected by two observers from each pair of adjacent sections. For the liver, the four holes in each image were marked by the same two observers. Most of the evaluated methods do not allow direct application of transformations to coordinates but support re-applying them to another stack of images. Therefore, we stored the landmarks as images with four disks placed at the landmark locations, each consisting of red, green, blue or yellow pixels. Color is invariant to the applied transformations, allowing post-registration detection of the disks. The tissue, mask and landmark images were downsampled to different resolutions and stored as TIF. See Supplementary Methods for details.

### 2.2 Evaluation of reconstruction accuracy

#### 2.2.1 Target registration error

Pairwise target registration error (TRE) (Fitzpatrick *et al.*, 1998), a direct measure of registration accuracy (Rohlfing, 2012), was quantified for each pair of adjacent sections. From the landmark images, we detected each landmark based on the colors of the disks and obtained their coordinates as the centroids of the detected pixels. For *N* pairs of sections, TRE was measured for each point (*j* = {1, 2, 3, 4}) and section pair (*i* = {1, 2,…, *N*}) as:

(1) $${TRE}_{j,i}=\left\|X_{j,i}-X_{j,i+1}\right\|$$

that is, the Euclidean distance between the location ***X*_*j,i*_** of point *j* on the section *i* and the location of the corresponding point on section *i *+* *1.

#### 2.2.2 Accumulated error

Accumulated target registration error (ATRE) was calculated to quantify distortion accumulated through the stack, referred to as ‘the banana problem’ (Malandain *et al.*, 2004) or ‘the shear effect’ (Hughes *et al.*, 2013). Each landmark of the prostate dataset is only present on two consecutive sections and pairwise errors on different sections should thus be independent of each other. However, in the presence of accumulated errors, the error vectors on nearby sections are correlated (Beare *et al.*, 2008). We quantified this effect by treating the displacement of each landmark (*j* = {1, 2, 3, 4}) for each pair of sections (*i* = {1, 2,…, *N*}) in vector form as $X_{j,i}-X_{j,i+1}$ and averaging the four vectors to obtain the mean displacement of each entire section. We then computed the cumulative sum of these mean vectors, proceeding from section 1 to section *N*. For section *k*, ATRE was defined as the Euclidean norm of the cumulative displacement vector:

(2) $${ATRE}_{k}=\|\sum_{i=1}^{k}\sum_{j=1}^{4}\frac{X_{j,i}-X_{j,i+1}}{4}\|$$

For the liver, a more direct quantification of ATRE was possible due to the landmarks extending through the sample. Ideally, the landmarks should lie on four parallel lines. In practice, parallelism could be violated due to slight movement of the sample between repeated applications of the laser. In a distorted volume, the landmarks deviate from the linear trajectories when proceeding through the stack. To measure this, we fitted a line in 3D to each of the four series of landmarks, minimizing mean squared error on the image plane. ATRE was then quantified for section *i* and landmark *j* as the Euclidean distance between the location of the landmark ***X*_*j,i*_** and that of the fitted line ***Y*_*j,i*_**, on the image plane:

(3) $$A{TRE}_{j,i}=\left\|X_{j,i}-Y_{j,i}\right\|$$

#### 2.2.3 Tissue shrinkage and overlap

As certain reconstruction methods tend to shrink the tissue, relative change in tissue area (ΔA-%) was computed based on the tissue masks for each section. Overlap was quantified based on the masks for each section pair using the Jaccard index (Rohlfing, 2012). The Jaccard index can be considered a quality measure for pixel-wise metrics, as computing them for a pair of sections with little overlap can provide misleading results. Let *A* denote the set of tissue pixels of section *i* and *B* the set of tissue pixels of section *i *+* *1. The Jaccard index is defined as:

(4) $${Jaccard}_{i}=\frac{\left|A\cap B\right|}{\left|A\cup B\right|}$$

#### 2.2.4 Pixel-wise similarity

For each section pair, we evaluated the similarity of corresponding pixels. After conversion to grayscale we computed the following measures: root mean squared error (RMSE), normalized cross correlation (NCC), mutual information (MI) and normalized mutual information (NMI) (Studholme *et al.*, 1999). Only the set of overlapping tissue pixels *A*∩*B* was considered. These indirect metrics provide information from the entire tissue area and complement the TRE evaluation.

#### 2.2.5 Reconstruction smoothness

We quantified the smoothness of the reconstruction using contrast *f_2_* and correlation *f_3_* based on gray-level co-occurrence matrices (GLCMs) (Cifor *et al.*, 2011; Gaffling *et al.*, 2015; Haralick and Shanmugam, 1973). Low contrast and high correlation indicate a smooth reconstruction. We formed the GLCM for each pair of grayscale images based on pixels *A*∩*B* and summed them to obtain a single GLCM for the whole volume.

### 2.3 3D reconstruction

- LS: Least-squares fitting of an affine transformation to the landmarks was implemented in MATLAB R2016b. The result is in principle unaffected by error accumulation (Xu *et al.*, 2015).
- OPT: Optimization-based reconstruction implemented in MATLAB R2016b was used to estimate pairwise affine transformations by minimizing the value of pixel-wise MSE.
- SIFT: Feature-based reconstruction was performed by computing SIFT keypoints (Lowe, 2004) for each image pair, establishing putative matches and robustly fitting an affine transformation to the point pairs (Fischler and Bolles, 1981). We used the RegisterVirtualStackSlices (Arganda-Carreras *et al.*, 2006) implementation in Fiji, also used as an initial step in RVSS and ESA.
- HSR: HyperStackReg v. 5 (Ved P. Sharma, Albert Einstein College, https://sites.google.com/site/vedsharma/imagej-plugins-macros/hyperstackreg) was run in Fiji to perform reconstruction using affine transformations.
- RVSS: Elastic reconstruction based on the bUnwarpJ algorithm, which is a combination of SIFT and optimization based methods, was applied using the RegisterVirtualStackSlices plugin in Fiji.
- ESA: The algorithm implemented in the ElasticStackAlignment plugin (Saalfeld *et al.*, 2012) was run via the TrakEM2 package (Cardona *et al.*, 2012) in Fiji to perform elastic reconstruction based on a combination of SIFT and optimization methods.
- MIM: Medical Image Manager, trial v. 0.94, was applied using images subsampled by a factor of 4 (magnification of 5×) as input. Sections 130 and 24 were used as references for the prostate and liver, respectively. We varied the initial magnification (0.3125×, 0.625×, 1.25× or 2.5×) and the number of non-rigid levels (1, 2, 3 or 4), thus modifying the image resolution used.
- Voloom: Trial v. 2.7.1 was used for elastic 3D reconstruction.

Fiji (Schindelin *et al.*, 2012; Schneider *et al.*, 2012) (v. 1.51h) plugins were run via ImageJ-MATLAB interface (v. 0.7.1) (Hiner *et al.*, 2016). Transformations were re-applied to the mask and landmark images. Output was saved as TIF. See Supplementary Methods for details.

### 2.4 Parameter optimization

In the case of MIM, which had to be operated interactively, we evaluated each combination of tunable values by a parameter sweep. Tunable parameters of the other methods were optimized via Bayesian optimization (Shahriari *et al.*, 2016; Snoek *et al.*, 2012), which is well-suited for such problems, where the objective function is computationally expensive to evaluate, nonconvex, multimodal, and typically has low to moderate dimensionality. Bayesian optimization has been shown to perform favorably in comparison to other global optimization algorithms on benchmarking functions (Jones, 2001) as well as on real WSI data (Teodoro *et al.*, 2017). We used MATLAB’s *bayesopt* implementation (https://www.mathworks.com/help/stats/bayesian-optimization-algorithm.html) with mean pairwise TRE as the objective function. We utilized a Gaussian process model of the objective function and an automatic relevance determination (ARD) Matérn 5/2 kernel (Snoek *et al.*, 2012) with ‘expected-improvement-plus’ as the acquisition function (Bull, 2011). Reconstructions with output image dimensions over fivefold compared to the input due to extreme error accumulation were considered failures. The number of variables to optimize was 2 (OPT), 4 (SIFT), 7 (RVSS) or 15 (ESA). We first optimized SIFT alone and used the optimal values for the SIFT step of RVSS and ESA. See Supplementary Table S1 for descriptions of the parameters. The number of seed points was set to twice the number of variables. We ran 30 iterations for OPT due to its simple objective function (Kartasalo *et al.*, 2016) and 100 iterations for the other tools. We used the prostate images subsampled by factors of 8 and 16, except for ESA, for which optimization was only feasible using the factor 16. Parameters optimized for ESA using the lower resolution were scaled to be used with the high resolution images. Computations were run on a workstation with Intel Xeon E5-1660 v3 3 GHz and 64 GB of RAM (low resolution) and a cluster node with Intel Xeon E5-2680 v3 2.5 GHz and 128 GB of RAM (high resolution).

## 3 Results

### 3.1 Effect of image resolution on evaluation metrics

First, we analyzed whether our metrics depend on image resolution (see Supplementary Results). TRE, ATRE, Jaccard and ΔA-% are essentially invariant to image resolution. They can be compared across different datasets and resolutions, as long as the accumulation of interpolation errors is avoided. RMSE, NCC, MI, NMI, *f_2_* and *f_3_* depend both on resolution and image content, and these metrics should thus only be compared within the same dataset and resolution. In all following analyses, we used images subsampled to pixel sizes of 7.36 and 3.68 µm, referred to as low and high resolution, respectively. The pixel sizes are close to the 5 µm section spacing and metrics computed from these images are not distorted by interpolation errors. Furthermore, we will only present RMSE as a measure of pixelwise similarity and *f_2_* as a measure of reconstruction smoothness due to their strong correlations with NCC, MI, NMI and *f_3_* (see Supplementary Table S1 for details).

### 3.2 Automated parameter tuning

Of the evaluated methods, LS, HSR and Voloom do not have tunable parameters. For OPT, SIFT, RVSS, ESA and MIM, we tuned the parameters automatically, minimizing the mean TRE computed for the prostate dataset. Parameter optimization took approximately 1500 hours in total to compute, producing 23 terabytes of data.

The optimization mostly converged close to the final solution in a handful of iterations (see Supplementary Results). By inspecting the variation in mean TRE values obtained during the process it is possible to reach a semi-quantitative view of the sensitivity of each method towards parameter adjustments. OPT and SIFT produced similar results for most parameter combinations while ESA, MIM and especially RVSS exhibited more sensitivity to parameter tuning.

We evaluated possible connections between accuracy and computation time, which might require the user to make a trade-off when selecting parameters (see Supplementary Results). The time taken by OPT varied only by a few minutes, except for the single inaccurate solutions where the parameters have not allowed proper convergence of the algorithm. For SIFT, there were no signs of a connection between accuracy and computation time. The differences in computation time between the fastest and slowest iterations of RVSS were roughly twofold and the fastest iterations were generally the ones with the highest error, indicating that minimizing the computation time of RVSS would sacrifice accuracy. In the case of ESA, the effect of parameter tuning was dramatic, leading to variation from approximately 12 min to more than 41 h. However, any clear relationship between computation time and accuracy was not observed.

### 3.3 Comparison of algorithms based on the prostate dataset

Results for the prostate dataset are listed in Table 1. The TRE values of LS based on landmarks by the two observers (LS1 and LS2) establish a baseline of accuracy. The case where the same landmarks were used for reconstruction and for calculating errors (LS1) is an optimistic estimate, representing the best accuracy reachable using an affine model. The errors calculated based on landmarks not used for reconstruction (LS2) represent a more realistic estimate of the accuracy of LS, serving as a cross-validation experiment between the two observers. The discrepancy between the optimistic and cross-validation results indicates that the LS solutions represent overfitting to the landmarks. Therefore, any methods with accuracy approaching LS can be regarded as highly accurate, since the other methods are not provided with any information concerning the landmarks. The systematic difference between TRE and ATRE calculated based on the two sets of landmarks (see Supplementary Table S1) is due to the fact that the two observers were free to select different landmarks and the error is generally not constant over the entire tissue section. However, using either set of landmarks leads to the same conclusions regarding the relative accuracy of the methods, confirmed by linear correlation coefficients of approximately 0.999 for mean TRE, 0.995 for maximum TRE, 0.888 for mean ATRE and 0.901 for maximum ATRE between the two sets of landmarks for the low resolution reconstructions. This also holds for the high resolution with corresponding values of 0.999, 0.986, 0.894 and 0.922. This indicates that even though four landmarks per section pair represent a relatively sparse sampling of the entire tissue section area, this number of landmarks is sufficient for reliable error estimation.

**Table 1.** Evaluation results for the prostate data at low (top) and high resolution (bottom)

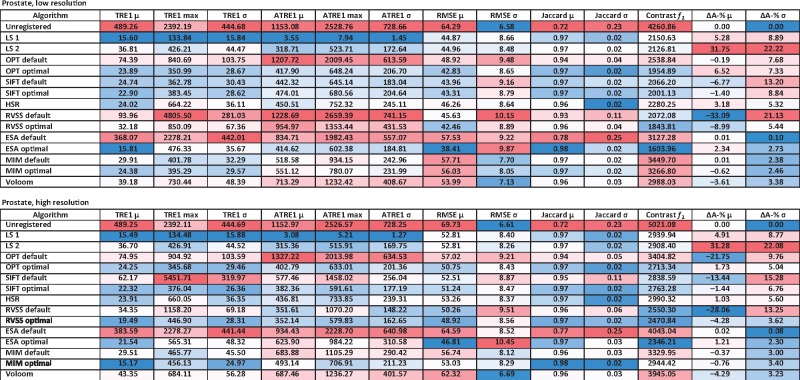

*Note*: Results for the unregistered images, LS based on landmarks by observer 1 (LS1) or 2 (LS2) and the automated methods (OPT, SIFT, HSR, RVSS, ESA, MIM, Voloom) using default or optimized parameters. Mean (μ), maximum (max) and standard deviation (σ) over all sections are shown. TRE and ATRE based on landmarks by observer 1 are in μm. In the online version, columns with TRE, ATRE, RMSE, *f2* and ΔA-% are colored from low (blue) to high values (red). Columns with Jaccard are colored from high (blue) to low values (red). (Color version of this table is available at *Bioinformatics* online.)

All methods benefited from parameter tuning on both image resolutions based on most of the metrics, using either set of landmarks for evaluation (see Table 1 and Supplementary Results). Of the top three methods, MIM and RVSS obtained better accuracy using high resolution images and ESA worked better on the low resolution images. ESA and MIM reached similar mean TRE values, slightly better than RVSS and approaching or exceeding the accuracy of LS. In terms of maximum TRE and ATRE, the three methods were comparable, but RVSS reached slightly lower ATRE than ESA or MIM. Among all tools, ESA and MIM also obtained the highest Jaccard index values. The RMSE and *f_2_* metrics do not allow comparison across different image resolutions and one should note that MIM’s output was always stored at the lower resolution for technical reasons. Considering these limitations, we can observe that ESA performed best in terms of these metrics on both image resolutions ahead of RVSS. Changes in tissue area introduced by ESA, MIM and RVSS were moderate. Behind the top three, most other tools reached accuracy comparable to each other. The worst results were obtained using default parameters and for some methods, most notably ESA and RVSS, they were even comparable to the unregistered original images.

**Fig. 2.**
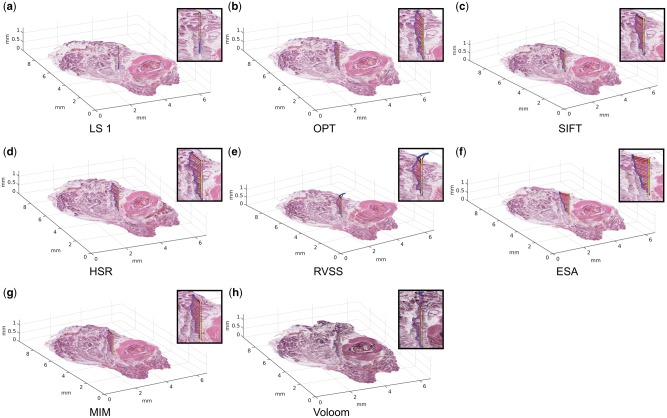
Reconstructions using (**a**) LS based on landmarks by observer 1, (**b**) OPT, (**c**) SIFT, (**d**) HSR, (**e**) RVSS, (**f**) ESA, (**g**) MIM and (**h**) Voloom. Optimized parameters and the most suitable resolution were used for each method. The dots represent the trajectory of accumulated target registration error from section to section. The horizontal lines indicate the direction and magnitude of the cumulative mean displacement of each section relative to the ideal error-free trajectory (vertical line). Magnified views are shown next to each reconstruction. Viewing the high-resolution color version of the Figure online is recommended. (Color version of this figure is available at *Bioinformatics* online.)

Visual examination in 3D revealed differences in the geometry of the reconstructions formed using each of the methods (Fig. 2). Compared to the undistorted reference (LS1), the distortions introduced by OPT, SIFT, HSR, ESA and MIM were a manifestation of the typical ‘banana-into-cylinder’ issue. This gradual straightening of curved structures is most clearly seen here in the displacement of the urethra at the top of the stacks. As indicated by the numerical ATRE values, the overall magnitude of this effect was rather similar across the tools. The distortions caused by RVSS and Voloom were more complex, representing clockwise twisting of the sample when seen from the top.

### 3.4 Comparison of algorithms based on the liver dataset

Results for the liver dataset are listed in Table 2. The four artificial landmarks were annotated by both observers and the two sets of TRE and ATRE values can be treated as replicates. This is reflected by linear correlation coefficients of approximately one (ranging from 0.99993 to 0.99998) for mean TRE, maximum TRE, mean ATRE and maximum ATRE calculated based on the two sets of landmarks (see Supplementary Table S1). In this case, LS thus represents an optimistic estimate of the accuracy reachable with a global affine model. Compared to the prostate sample, this dataset is more challenging to reconstruct due to the more homogeneous appearance of the tissue and the presence of deformations such as folded and torn tissue. This is reflected by the metrics, which generally indicate higher errors, except for RMSE and *f_2_* which are lower due to the more homogeneous image content. Ideally, it would be convenient to process different datasets without having to readjust parameters. With this in mind, we reused the parameters optimized for the prostate dataset, treating the evaluation on the liver dataset as an independent validation experiment. Based on most metrics, the optimized parameters generally resulted in an improvement over the default parameters also when applied to the liver dataset (see Table 2 and Supplementary Results).

**Table 2.** Evaluation results for the liver data at low (top) and high resolution (bottom)

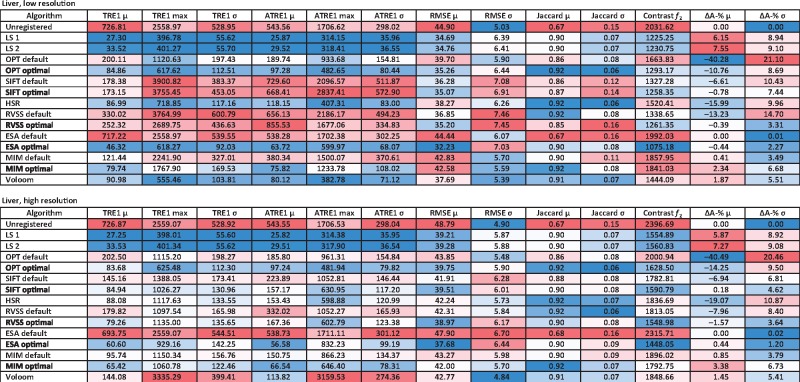

*Note*: Results for the unregistered images, LS based on landmarks by observer 1 (LS1) or 2 (LS2) and the automated methods (OPT, SIFT, HSR, RVSS, ESA, MIM, Voloom) using default or optimized parameters. Mean (μ), maximum (max) and standard deviation (σ) over all sections are shown. TRE and ATRE based on landmarks by observer 1 are in μm. In the online version, columns with TRE, ATRE, RMSE, *f2* and ΔA-% are colored from low (blue) to high values (red). Columns with Jaccard are colored from high (blue) to low values (red). (Color version of this table is available at *Bioinformatics* online.)

As with the prostate, the lowest TRE values among the automated methods were achieved by ESA on the lower resolution and MIM on the high resolution data with RVSS being the third best method. The other methods reached TRE values comparable to each other. In terms of maximum TRE and ATRE, the conclusion was less clear. Voloom performed better on the lower resolution, reaching a maximum TRE second only to LS, while ESA and OPT also reached comparable values. On this dataset, MIM suffered from larger maximum errors compared to the higher quality prostate sample. The lowest mean ATRE values among all automated methods were obtained by ESA, MIM and Voloom, while in terms of maximum ATRE Voloom was superior to ESA and MIM. ESA was the top method in terms of RMSE and *f_2_*, and MIM obtained the highest Jaccard index. Again, the poorest results were obtained when using the default values of tunable parameters.

**Fig. 3.**
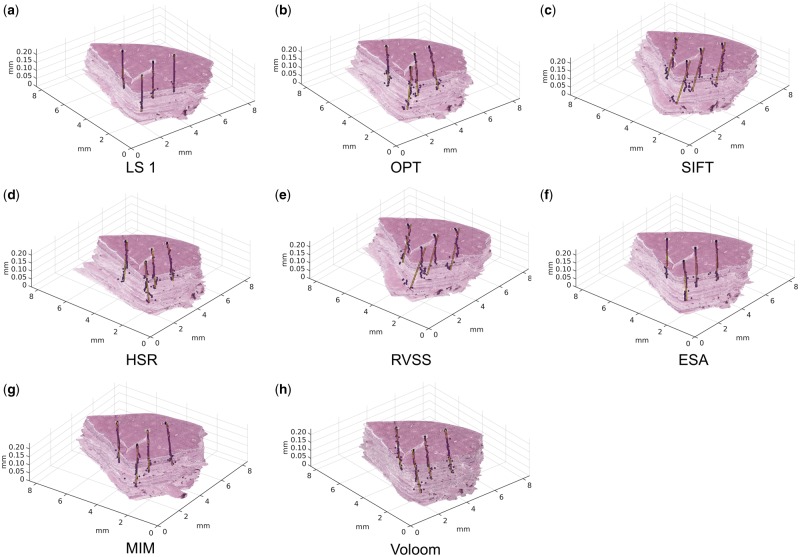
Reconstructions using (**a**) LS based on landmarks by observer 1, (**b**) OPT, (**c**) SIFT, (**d**) HSR, (**e**) RVSS, (**f**) ESA, (**g**) MIM and (**h**) Voloom. Optimized parameters and the most suitable resolution were used for each method. The locations of the four landmark points on each section are indicated with dots, shown together with lines of best fit to each of the four series of points. Note that the scale of the vertical axis is different from the horizontal axes in the visualization. Viewing the high-resolution color version of the Figure online is recommended. (Color version of this figure is available at *Bioinformatics* online.)

Visualization in 3D supported the numerical results (Fig. 3). ESA, MIM and Voloom formed reconstructions with landmarks concentrated on four roughly parallel lines as expected, but some distortion is visible at the bottom part of the stack reconstructed by MIM. These kind of distortions were more severe in the case of OPT, SIFT, HSR and RVSS.

## 4 Discussion

Based on this study, methods utilizing locally varying transformations (ESA, MIM, RVSS, Voloom) were superior to those constrained to global affine models (OPT, SIFT, HSR). ESA was the only method to consistently outperform or match the other approaches on two datasets based on the majority of metrics. In the case of the higher quality prostate dataset, differences in accuracy between the tools were rather subtle. All three top-performing methods on this dataset incorporate an elastic transformation model: MIM and RVSS use a B-spline grid and ESA is based on a piecewise linear mesh. While methods relying on a global transformation model also performed reasonably well, the additional accuracy offered by elastic transformations could be crucial when microstructure at the cellular scale is of interest. In the case of the liver sample, more profound differences between the methods were observed, likely due to the more challenging tissue content and the presence of deformations, which cannot be compensated for using a global model. ESA, MIM and Voloom stood out from the other methods. While Voloom appeared to be less accurate on average compared to ESA and MIM based on mean TRE, it demonstrated the lowest maximum and accumulated errors of all automated methods, indicating capability to avoid propagation of errors even in the presence of considerable deformations. The ability of the algorithms to tolerate such deformations is a significant benefit. Due to the mostly manual nature of histological sectioning and brittleness of the thin tissue sections, deformations in the form of folds and tears often occur. This challenge is especially encountered in 3D histology, when uninterrupted sequences of sections are desired.

Another important property of algorithms to consider is sensitivity to adjustable parameters. Even an algorithm that produces highly accurate results with a carefully selected set of parameter values will be useless if the user has little chance of finding this set of values. Comparing algorithms from this perspective is difficult. Each algorithm has a different set of parameters and the range of values to evaluate has to be selected for each parameter, which can in turn affect the amount of variation observed in the results. Nevertheless, this study still provides a semi-quantitative view of the sensitivity of the studied algorithms against parameter adjustments. Of the evaluated methods, LS, HSR and Voloom are the most convenient due to their lack of tunable parameters. OPT and SIFT also produced similar results with most parameter values. The results produced by ESA varied greatly depending on parameters, but we discovered numerous combinations leading to almost optimal results. In the case of MIM, there are only a handful of tunable parameters and they are relatively easy to tune. Moreover, ESA and MIM appear to be well-behaving in the sense that parameters optimized for the prostate dataset also suited the liver dataset. In contrast, RVSS was found to be difficult to optimize and even though its accuracy using optimized settings was close to ESA and MIM on the prostate dataset, reaching this level of accuracy without automated parameter tuning would be challenging.

An open question common to all of the methods is how image resolution affects reconstruction accuracy. A pixel size close to the section spacing is often recommended (Amunts *et al.*, 2013; Braumann *et al.*, 2005; Dauguet *et al.*, 2007; Ju *et al.*, 2006; Kartasalo *et al.*, 2016; Saalfeld *et al.*, 2012) based on the assumption that objects smaller than this are only visible on a single section and are thus not useful for registration, and may even introduce errors (Beare *et al.*, 2008). However, suitably oriented elongated structures such as blood vessels can be observed on several sections even if their diameter on the image plane is smaller than the section spacing. In principle, some algorithms might thus benefit from a smaller pixel size. We evaluated reconstruction accuracy using pixel sizes of 3.68 and 7.36 µm. Based on the rule of thumb above, it is unclear which one of these should be preferred given a section spacing of 5 µm. Our results indicate that using a pixel size close to the section spacing is a reasonable starting point, but the optimal image resolution depends on the algorithm and also somewhat on the image content. Furthermore, we cannot rule out the possibility that algorithms which performed better on the high resolution images, most notably MIM, might benefit from an even smaller pixel size. In conclusion, the image resolution thus needs to be selected experimentally for each application and algorithm.

The two samples selected for this study are markedly different in their histological composition. The fact that the top methods performed well on both the prostate and the liver dataset without any retuning of parameters indicates that these methods are not overly sensitive to tissue appearance, and that the results obtained in this study are not specific to a single dataset. However, some variation in the relative performance of the algorithms on the two datasets was still observed. Thus, collecting and annotating additional datasets representing diverse tissue types and other histological stainings, such as immunohistochemistry, remains an important goal for future studies.

While we evaluated a comprehensive set of methods for 3D histology, it might be worthwhile to adapt general-purpose image registration algorithms to this context. Another opportunity, not supported by any of the methods here, could be the exploitation of additional data obtained e.g. by magnetic resonance imaging or in the form of blockface images (Amunts *et al.*, 2013; Casero *et al.*, 2017; Dauguet *et al.*, 2007; Gibson *et al.*, 2013; Johnson *et al.*, 2010; Stille *et al.*, 2013). Furthermore, although advances in image acquisition and processing have enabled the first steps towards 3D histology, sample preparation still constitutes a significant bottleneck. In the future, emerging technologies for automated sample preparation (Onozato *et al.*, 2011) or integrated sectioning and imaging (Li *et al.*, 2010; Ragan *et al.*, 2012) might potentially transform 3D histology into a high-throughput process.

## Supplementary Material

Supplementary Data
