# Supplementary material for: Comparative analysis of tissue reconstruction algorithms for 3D histology
Source: Bioinformatics. 2018 Apr 19;34(17):3013–21. doi: 10.1093/bioinformatics/bty210 (PMC6129300; doi:10.1093/bioinformatics/bty210)
Supplement: Supplementary Data [file bty210_supp.zip › bty210-suppl_data/supplementary_information.pdf]

Supplementary information

Comparative Analysis of Tissue Reconstruction Algorithms for 3D Histology

Kimmo Kartasalo, Leena Latonen, Jorma Vihinen, Tapio Visakorpi, Matti Nykter, Pekka Ruusuvuori

1. Supplementary methods

1.1. Sample preparation

The prostate of a 14 month old male FVB/N mouse was fixed in PAXgene™ (PreAnalytiX GmbH, Hombrechtikon, Switzerland) and embedded in paraffin. The tissue block was sectioned through, with 3x3 5 µm sections used for hematoxylin-eosin (HE) staining, and every 10th section saved for other purposes.

The liver of a male FVB/N mouse was fixed in formalin and submerged in 70 % ethanol. The sample was placed on a glass slide for processing with a laser in order to introduce artificial landmarks into the otherwise homogeneous tissue. An SPI 70 W fiber laser with GSI scanner (f167 mm F-theta focal lens) was used with the following parameters: wavelength 1090 nm, pulse frequency 752 kHz and power 65 W. Each hole was produced by applying the laser for 100 ms 3-5 times until the entire sample had been penetrated. Four holes were successfully introduced into the sample. Finally, the sample was embedded in paraffin, serially cut to 5 µm sections and HE stained.

1.2. Image acquisition

Slides were scanned at 20x using a Zeiss Axioskop40 microscope (Carl Zeiss MicroImaging, NY, USA) with a CCD color camera (QICAM Fast; QImaging, Canada) and a motorized specimen stage (Märzhäuser Wetzlar GmbH, Germany). Image acquisition was controlled by the Surveyor system (Objective Imaging, UK). Uncompressed output was converted by JP2 WSI Converter to JPEG2000 (Tuominen and Isola, 2010). Pixel size was 0.46 µm. The prostate and liver datasets contained 260 and 47 RGB images, respectively, with one section per image.

1.3. Image preprocessing and annotation

Further processing was performed in MATLAB R2016b (The MathWorks Inc., Natick, MA, USA). Tissue was segmented from background by downsampling the images by a factor of 8, computing the HSV transform and applying a threshold of 0.25 t_{Otsu} on the S component, where t_{Otsu} is the value obtained by Otsu's method (Otsu, 1979). The resulting binary mask was processed by dilation, filling of holes and erosion with a disk element (radius 4 pixels) to obtain smooth regions. Objects with less than 400 000 pixels were removed. Non-tissue pixels were assigned an intensity of 255 to produce a white background.

A total of 2448 landmarks were manually annotated. In the prostatic tissue, four corresponding points preferably at the centers of bisected nuclei were selected by two observers from each pair of adjacent sections. For the liver, the four holes in each image were marked by the same two observers with an ellipse tool, using the ellipse's centroid as the final coordinate. Most of the evaluated methods do not allow exporting transformations, preventing their direct application to coordinates. However, it is possible to re-apply the transformations to another stack of images. Therefore, we stored the landmarks as RGB images with four disks (radius 150 pixels) placed at the landmark locations, consisting of either red, green, blue or yellow pixels. Color is invariant to the applied transformations, allowing post-registration detection of the disks.

The tissue images, masks and landmark images were stored in TIF format. Downsampled versions were obtained via bilinear (tissue images) or nearest neighbor interpolation (masks and landmark images).

1.4. Least-squares reference 3D reconstruction

The reference LS method was implemented in MATLAB R2016b by fitting a least-squares optimal affine transformation to the landmark points for each pair of images using the fitgeotrans function. The pairwise transformations were concatenated serially and applied via bilinear (tissue images) or nearest-neighbor (masks and landmark images) interpolation using the imwarp function. Output images were saved in TIF format. As the landmarks represent bisected nuclei appearing on only two consecutive sections, the reference reconstruction is in principle unaffected by error accumulation over multiple sections (Xu et al., 2015).

1.5. Optimization-based 3D reconstruction

The OPT method was implemented in MATLAB R2016b using the imregtform function. Regular step gradient descent was used to estimate affine transformations relating each pair of images by minimizing the value of pixel-wise MSE. Optimization was initialized with a simple translation computed based on the tissue section centroids. The pairwise transformations were concatenated serially and applied via bilinear (tissue images) or nearest-neighbor (masks and landmark images) interpolation using the imwarp function. Output images were saved in TIF format.

1.6. Feature-based 3D reconstruction

The SIFT method was realized by computing SIFT keypoints (Lowe, 2004) for each image pair, establishing putative corresponding points and robustly estimating a transformation from the keypoint pairs for each image pair (Fischler and Bolles, 1981). We used the ImageJ/Fiji (Schindelin et al., 2012; Schneider et al., 2012) (version 1.51h) implementation in the RegisterVirtualStackSlices plugin (Arganda-Carreras et al., 2006). The same underlying implementation is also used as an initial step in the elastic reconstruction methods of the RegisterVirtualStackSlices and ElasticStackAlignment (Saalfeld et al., 2012) plugins. The transformations applied to the tissue images were saved in XML format and re-applied to the masks and landmark images using the TransformVirtualStackSlices plugin (http://imagej.net/Transform_Virtual_Stack_Slices). Output images were saved in TIF format. The plugins were used via a jython script through the ImageJ-MATLAB interface (Hiner et al., 2016), version 0.7.1.

1.7. HyperStackReg 3D reconstruction

HyperStackReg, version 5, written by Ved P. Sharma (Albert Einstein College, New York, https://sites.google.com/site/vedsharma/imagej-plugins-macros/hyperstackreg) as an extension to StackReg (Thevenaz et al., 1998) was run via a macro in ImageJ/Fiji (Schindelin et al., 2012; Schneider et al., 2012) (version 1.51h). The RGB tissue images and an additional channel corresponding to the mask or one of the landmark image channels were stacked into four-channel hyperstacks. The hyperstacks were processed by HSR using only the tissue image channels for estimating the affine transformations while applying the final transformations to all channels. The process was repeated for each of the additional channels in order to apply the transformations to the masks and all landmark image channels. Output images were saved in TIF format

1.8. RegisterVirtualStackSlices 3D reconstruction

The RegisterVirtualStackSlices plugin, based on the bUnwarpJ algorithm (Arganda-Carreras et al., 2006), for ImageJ/Fiji (Schindelin et al., 2012; Schneider et al., 2012) (version 1.51h) was used for 3D reconstruction. The transformations applied to the tissue images were saved in XML format and re-applied to the masks and landmark images using the TransformVirtualStackSlices plugin (http://imagej.net/Transform_Virtual_Stack_Slices). Output images were saved in TIF format. The plugins were used via a jython script through the ImageJ-MATLAB interface (Hiner et al., 2016), version 0.7.1

1.9. ElasticStackAlignment/TrakEM2 3D reconstruction

The ElasticStackAlignment plugin (Saalfeld et al., 2012), incorporated into the TrakEM2 package (Cardona et al., 2012) for ImageJ/Fiji (Schindelin et al., 2012; Schneider et al., 2012) (version 1.51h) was used for 3D reconstruction. The transformations were re-applied to the masks and landmark images by saving the TrakEM2 project as an XML file, modifying the filenames in the XML and by opening the modified project file in TrakEM2. Output images were saved in TIF format. The plugin was used via a jython script through the ImageJ-MATLAB interface (Hiner et al., 2016), version 0.7.1

1.10. Medical Image Manager 3D reconstruction

Medical Image Manager (HeteroGenius Ltd, Leeds, UK), trial version 0.94, was used for 3D reconstruction. Images subsampled by a factor of 4 (magnification of 5X) were used as input. Default values were used for the padding (10%), spline patch size (256x256), number of iterations (one for each level) and the spline grid sizes (3x3 for level 1, 6x6 for level 2, 9x9 for level 3, 12x12 for level 4), as suggested by the software provider. Sections 130 and 24 were used as reference sections for the prostate and liver, respectively. Different combinations of initial and final magnification were tested by varying the initial magnification (0.3125X, 0.625X, 1.25X or 2.5X) and the number of non-rigid levels (1, 2, 3 or 4), thus essentially modifying the image resolution used for registration. The transformations were re-applied to the masks and landmark images by saving the alignments as XML files, modifying the filenames and by uploading the modified XML files back to MIM. The reconstructed volumes were saved in MHD format at a subsampling factor of 16 and converted to a series of TIF images using the University of Leeds Volume Viewer version 14.01 (http://www.comp.leeds.ac.uk/drm/TDPW-VolumeViewer14.01.msi).

1.11. Voloom 3D reconstruction

Voloom trial version 2.7.1 (microDimensions GmbH, Munich, Germany) was used for 3D reconstruction. The non-rigid transformations were re-applied to the masks and landmark images by saving them as MDV files, modifying the filenames in the underlying XML files and by uploading the modified MDV files back to Voloom. Output images were saved in TIF format. The white background regions introduced at the edges of the mask images by Voloom were removed by setting their pixel values to 0.

2. Supplementary results

2.1. Effect of image resolution on the evaluation metrics

To understand whether the metrics employed in our benchmarking framework are invariant to the in-plane resolution of the images, we computed their values for three reconstructed volumes representing different degrees of misalignment, consisting of images downsampled using subsampling ratios of 2, 4, 8, 16 and 32. The LS result was used as an example of an accurately reconstructed volume. To generate a slightly misaligned volume, we translated each of the images in the LS reconstruction along each dimension by a randomly selected number of pixels, sampled from uniform distributions in the interval [-100, 100], corresponding to [-46, 46] µm. To generate a volume with severe misalignment, we sampled the translation from the interval [-1000, 1000], corresponding to [-460, 460] µm. We applied the same procedure to the prostate and liver datasets. Since the LS reconstruction was formed based on the coordinates of landmark points marked at full resolution and these coordinates were simply scaled accordingly for registering the downsampled images, the alignment of the tissue sections is identical in all of the subsampled volumes. Therefore, any differences in the results computed using different image resolutions only represent the direct effect of image resolution on the metrics.

Based on the results, presented in Figure S1 and Figure S2, the landmark-based TRE and ATRE metrics are essentially invariant to image resolution. Differences in the mean TRE between downsampling ratios of 2 to 16 are less than 100 nm and even at the coarsest resolution, the difference to the higher resolution values of approximately 0.5 µm is comparable to a single pixel at full resolution. In the case of ATRE, the accumulation of interpolation errors leads to a largely overestimated error value for the images of the prostate dataset at the coarsest resolution, while the changes in the metric observed at all the other resolutions are in the range of a few micrometers or 1-2 pixels. Also the Jaccard tissue overlap metric and the tissue shrinkage metric ΔA-% were essentially invariant to changes in image resolution, except for small differences due to interpolation errors. This behavior was expected since these metrics quantify the relative overlap between adjacent tissue sections and the relative change in tissue area of each section and thus do not depend on the absolute number of pixels. Thus, the TRE, ATRE, Jaccard and ΔA-% metrics can be compared across different datasets and resolutions, as long as the resolution is sufficient to avoid the accumulation of interpolation errors. The pixelwise metrics and the contrast and correlation metrics appear heavily dependent on image resolution and tend to improve with increased downsampling of the images. This is a logical consequence due to the fact that all of these metrics quantify similarity (NCC, MI, NMI, f_3) or dissimilarity (RMSE, f_2). Loss of details due to downsampling increases the similarity, or equivalently decreases the dissimilarity, of corresponding pixels. There is also an interesting difference between the two datasets: the different magnitude of the error in the slightly and severely misaligned volumes is correctly captured by the TRE, ATRE and Jaccard metrics, and this also holds for the pixelwise metrics in the case of the liver dataset, but not in the case of the prostate dataset. For the liver dataset, also the pixelwise metrics reflect the true situation where the accurate volume and the slightly misaligned volume contain much smaller errors than the severely misaligned volume. In other words, the behavior of pixelwise similarity or dissimilarity metrics depends both on image resolution and the appearance of the tissue, and the values of these metrics should thus only be compared within the same dataset and resolution.

2.2. Effect of parameter selection on reconstruction quality

Automated parameter optimization mostly converged close to the final solution in a handful of iterations (see Figure S3). By inspecting the variation in mean TRE values obtained during the process it is possible to reach a semi-quantitative view of the sensitivity of each method towards parameter adjustments. OPT (std = 30.3 µm for low resolution, std = 30.6 µm for high resolution) and SIFT (std = 6.3 µm for low resolution, std = 3.4 µm for high resolution) produced similar results for most parameter combinations while RVSS (std = 205.8 µm for low resolution, std = 193.6 µm for high resolution) and ESA (std = 166.8 µm for low resolution) exhibited more sensitivity to parameter tuning. The reconstruction failed on a number of iterations in the case of SIFT (25 and 19 failures on low and high resolution, respectively) and RVSS (17 and 8 failures). In the case of MIM and low resolution, the mean TRE values obtained were 24.4 µm, 29.9 µm, 46.7 µm, 84.9 µm, 298.0 µm and 445.9 µm, leading to a standard deviation of 175.7 µm. Additional

parameter combinations enabled by the higher resolution produced values of 15.2 µm, 15.6 µm, 29.5 µm, 504.9 µm, increasing the standard deviation to 191.8 µm.

The results in terms of main metrics obtained for the prostate dataset using optimized and default settings, meaning sensible baseline parameter values selected based on available documentation, are tabulated in Table 1. Full results with the values of all metrics are presented in Table S1. All of the methods benefited from the parameter tuning, if assessed based on the improvements in mean TRE of 67.8% (OPT), 7.5% (SIFT), 65.8% (RVSS), 95.7% (ESA) and 18.5% (MIM) for the low resolution images or corresponding improvements of 67.6%, 64.1%, 43.3%, 94.4% and 48.6% for the high resolution data. We repeated the analysis using landmark points selected by a second observer, leading to corresponding values of 68.1%, 10.3%, 73.9%, 96.9% and 22.4% (low resolution) or 68.8%, 65.7%, 47.5%, 96.0% and 48.8% (high resolution). Overall, the quality metrics, including mean and maximum TRE and ATRE based on the two sets of landmarks, mean RMSE, Jaccard's index and the f_2 measure, improved by 54.7% ± 28.0% for OPT, 1.6% ± 15.9% for SIFT, 38.7% ± 32.9% for RVSS, 67.8% ± 25.0% for ESA and 5.3% ± 17.8% for MIM (mean ± standard deviation over different metrics) in the case of the low resolution prostate data. For the high resolution data, improvements of 54.8% ± 28.6% for OPT, 50.5% ± 35.3% for SIFT, 34.6% ± 28.6% for RVSS, 55.6% ± 28.5% for ESA and 27.2% ± 18.8% for MIM were observed. In the case of SIFT and the low resolution images, the overall improvement was accompanied by small increases in the mean and maximum ATRE. In the case of MIM, there was also a small increase in the mean ATRE computed based on the first set of landmarks and a larger increase in the maximum TRE computed based on the second set of landmarks. These effects were not observed when using the high resolution images, as the only detrimental effect caused by the parameter optimization was the minimal 0.15% increase in mean ATRE for RVSS.

One indication of the robustness of the reconstruction algorithms is the ability to apply them to another dataset consisting of different images without having to readjust any parameters. To evaluate the algorithms from this viewpoint, we applied the reconstruction methods to a series of sections from a murine liver using both default parameters and the parameters optimized for the prostate dataset, and compared the resulting 3D reconstructions. The resulting values of the main quality metrics are tabulated in Table 2. Full results with the values of all metrics are presented in Supplementary Table S1. In the case of the low resolution liver dataset, the mean TRE values obtained by OPT, SIFT, RVSS, ESA and MIM using optimized parameters were improved relative to the ones obtained using default parameters by 57.6%, 2.9%, 23.6%, 93.5% and 34.3%, respectively. For the high resolution images, the corresponding improvements were 58.7%, 41.5%, 55.9%, 91.3% and 31.7%. Using a second set of landmarks to repeat the analysis, the corresponding values were 56.5%, 2.9%, 23.7%, 93.1% and 34.6% (low resolution) or 57.6%, 40.8%, 54.3%, 90.6% and 30.8% (high resolution). The overall improvement over the quality metrics for the low resolution liver dataset was 39.8% ± 17.8% for OPT, -2.9% ± 16.2% for SIFT, 8.5% ± 22.4% for RVSS, 68.5% ± 23.3% for ESA and 28.1 ± 28.1% for MIM (mean ± standard deviation over different metrics). For the high resolution images, corresponding improvements of 39.4% ± 18.7% for OPT, 26.7% ± 14.5% for SIFT, 28.2% ± 25.0% for RVSS, 61.9% ± 25.6% for ESA and 22.7 ± 19.5% for MIM were observed. Thus, parameters optimized for one dataset can be useful also when applied to another dataset, even in the case of a different tissue type.

Supplementary references

Otsu,N. (1979) A threshold selection method from gray-level histograms. IEEE Trans.Syst.Man Cybern., 9, 62-66.

Tuominen,V.J. and Isola,J. (2010) Linking whole-slide microscope images with DICOM by using JPEG2000 interactive protocol. J.Digital Imaging, 23, 454-462.

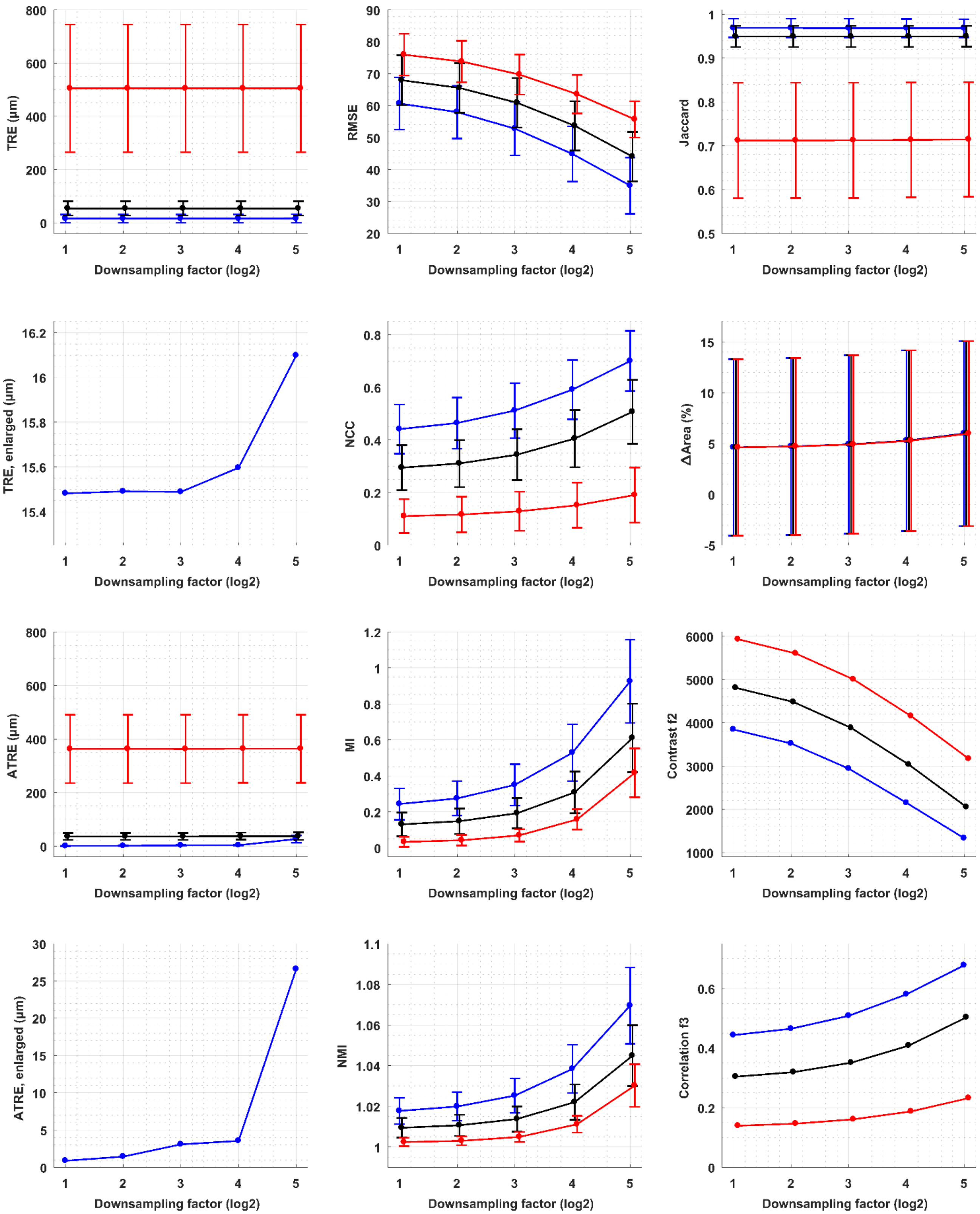

Figure S1: Evaluation metrics by resolution for the prostate dataset.

Values of quality metrics computed from the prostate dataset at different image resolutions obtained by downsampling the full-resolution images with factors 2, 4, 8, 16 and 32. The blue graphs represent an accurate reference volume, the black graphs represent a slightly misaligned volume and the red graphs represent a severely misaligned volume. The data points correspond to mean values over the entire stack (N = 259) and the one-sided lengths of the error bars correspond to one standard deviation. Error bars are not shown for the contrast f_2 and correlation f_3 measures, since a single value of these metrics is computed for the entire volume. Enlarged views of the TRE and ATRE graphs for the accurate reference volumes are shown with the error bars removed for clarity.

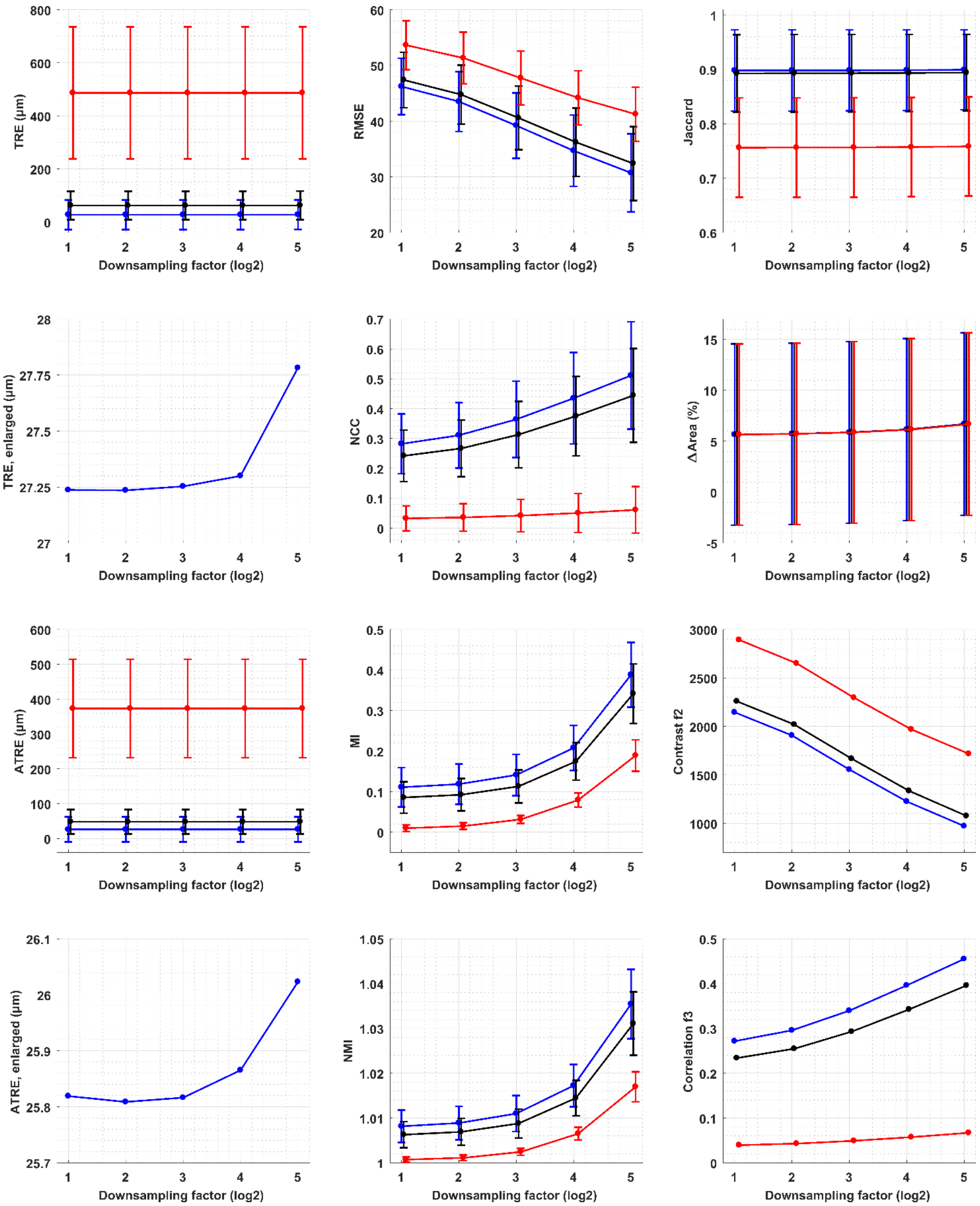

Figure S2: Evaluation metrics by resolution for the liver dataset.

Values of quality metrics computed from the liver dataset at different image resolutions obtained by downsampling the full-resolution images with factors 2, 4, 8, 16 and 32. The blue graphs represent an accurate reference volume, the black graphs represent a slightly misaligned volume and the red graphs represent a severely misaligned volume. The data points correspond to mean values over the entire stack (N = 47) and the one-sided lengths of the error bars correspond to one standard deviation. Error bars are not shown for the contrast f_2 and correlation f_3 measures, since a single value of these metrics is computed for the entire volume. Enlarged views of the TRE and ATRE graphs for the accurate reference volumes are shown with the error bars removed for clarity.

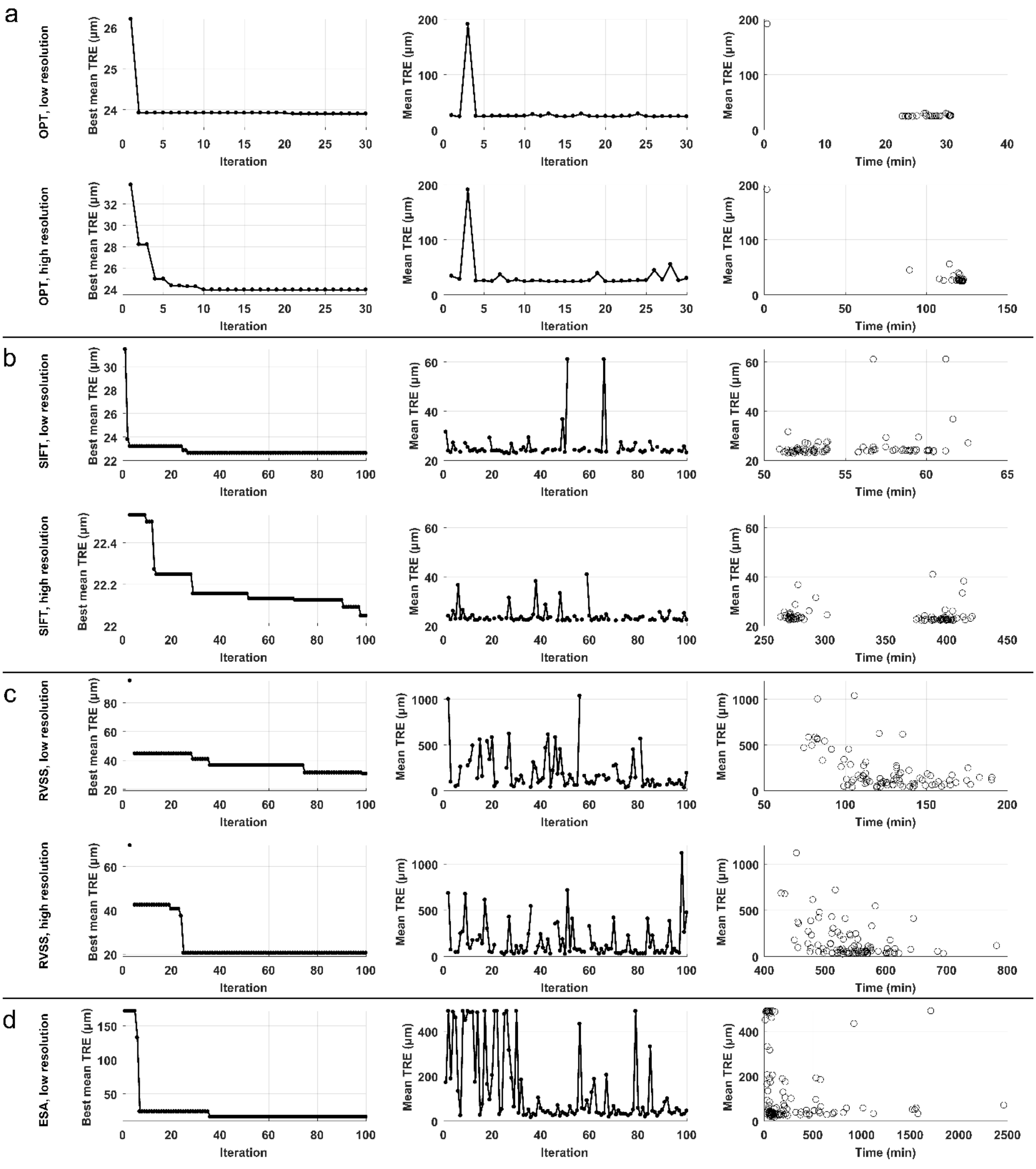

Figure S3: Automated parameter tuning.

Results of parameter optimization for (a) OPT, (b) SIFT, (c) RVSS and (d) ESA using low (top rows) or high (bottom rows) resolution. For ESA, only low resolution was used. Plotted on each row are the convergence curve of the lowest mean TRE observed by each iteration (left), the current mean TRE on each iteration (center) and mean TRE versus computing time for each iteration (right). The times reported for low and high resolution may not be directly comparable due to different hardware. Mean TRE values are in µm. Missing points correspond to failed iterations.
